# Therapeutic Effect of Anti-VEGF for Age-Related Macular Degeneration in the Untreated Fellow Eye

**DOI:** 10.1155/2018/8561895

**Published:** 2018-04-24

**Authors:** Hacer Isildak, Stephen G. Schwartz, Harry W. Flynn

**Affiliations:** ^1^Department of Ophthalmology, Bascom Palmer Eye Institute, University of Miami Miller School of Medicine, 3880 Tamiami Trail N., Naples, FL 34103, USA; ^2^Department of Ophthalmology, Bascom Palmer Eye Institute, University of Miami Miller School of Medicine, 900 NW 17th St., Miami, FL 33136, USA

## Abstract

Intravitreal injections of antivascular endothelial growth factor (anti-VEGF) agents have been reported to occasionally produce a therapeutic effect in the uninjected fellow eye. Here, three patients with bilateral neovascular age-related macular degeneration are presented. In all three patients, unilateral anti-VEGF injection resulted in bilateral reduction of macular thickness as measured by spectral domain optical coherence tomography.

## 1. Introduction

The use of antivascular endothelial growth factor (anti-VEGF) agents is standard treatment for neovascular age-related macular degeneration (AMD), diabetic macular edema (DME), and other disorders [1, 2]. Several studies have reported bilateral therapeutic effects following unilateral anti-VEGF injection in patients with AMD [3–5], DME [6–11], and other disorders.

Here, three patients with bilateral neovascular AMD are presented in which unilateral injection of anti-VEGF agents resulted in bilateral reduction of macular thickening as measured by spectral domain optical coherence tomography (SD-OCT). Institutional Review Board approval was not required for this study.

## 2. Case Reports

### 2.1. Patient 1

**Figure 1:**
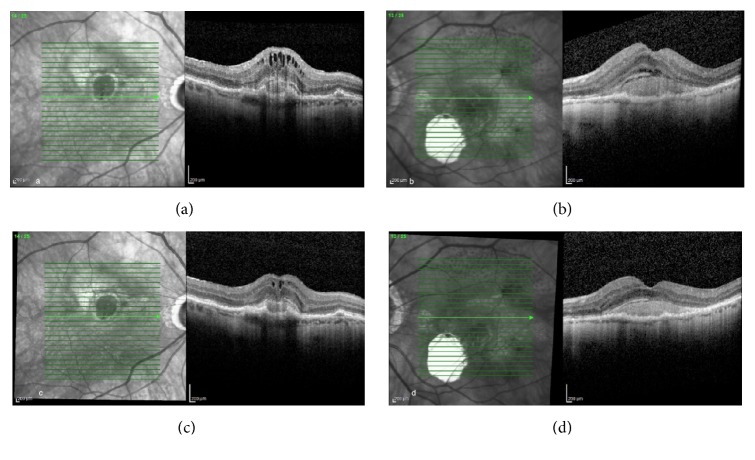
An 86-year-old male with bilateral neovascular age-related macular degeneration (AMD) ((a) and (b)). Nine days following bevacizumab injection OD, there was a reduction in intraretinal and subretinal fluid OU ((c) and (d)).

An 86-year-old male with bilateral neovascular AMD returned for scheduled followup. Best corrected visual acuity (BCVA) was 20/100 OD and 20/150 OS. SD-OCT revealed cystoid macular edema (CME) OD and solid pigment epithelial detachment (PED) with subretinal fluid OS. The patient was treated with an intravitreal injection of bevacizumab 1.25 mg (Avastin, Genentech, South San Francisco, CA) OD. Nine days following the injection, the patient returned urgently, complaining of metamorphopsia OD. Visual acuity (VA) was 20/60 OD and 20/150 OS, with decreased central macular thickness in each eye (OU) (Figure 1).

### 2.2. Patient 2

**Figure 2:**
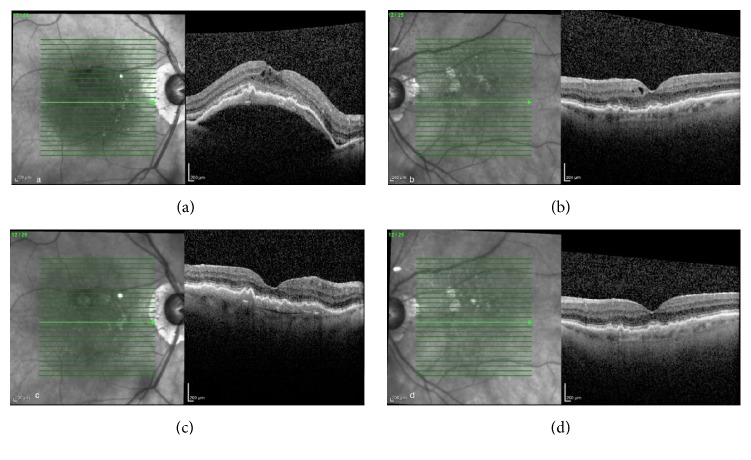
An 86-year-old female with neovascular AMD OU ((a) and (b)). Five weeks following aflibercept injection OD, there was a reduction in intraretinal and subretinal fluid OU ((c) and (d)).

An 86-year-old female with bilateral neovascular AMD returned for scheduled followup. VA with pinhole was 20/70 OD and 20/50 OS. SD-OCT revealed CME and serous PED OD as well as CME OS. The patient was treated with an intravitreal injection of aflibercept 2 mg (Eylea, Regeneron, Tarrytown, NY) OD. Five weeks later, VA with pinhole was 20/40 OU with decreased central macular thickness OU (Figure 2).

### 2.3. Patient 3

**Figure 3:**
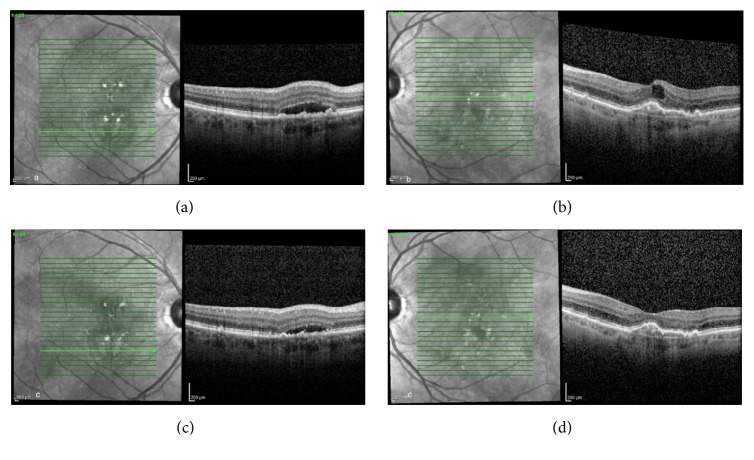
A 74-year-old male with bilateral neovascular AMD ((a) and (b)). Six weeks following intravitreal injection of ranibizumab OS, there was a reduction in intraretinal and subretinal fluid OU ((c) and (d)).

A 74-year-old male with bilateral neovascular AMD returned for scheduled followup. VA with pinhole was 20/25 OD and 20/40 OS. SD-OCT revealed PED OU. The patient was treated with an intravitreal injection of ranibizumab 0.5 mg (Lucentis, Genentech, South San Francisco, CA) OS. At scheduled followup six weeks later, BCVA was 20/20 OD and 20/50 OS with decreased central macular thickness OU (Figure 3).

## 3. Discussion

In most patients, unilateral injection of anti-VEGF does not cause a clinically significant effect in the fellow eye. For example, in two large prospective randomized clinical trials with neovascular AMD in one eye and nonneovascular AMD in the fellow eye (Minimally Classic/Occult Trial of the Anti-VEGF Antibody Ranibizumab in the Treatment of Neovascular Age-Related Macular Degeneration [MARINA] and Anti-VEGF Antibody for the Treatment of Predominantly Classic Choroidal Neovascularization in Age-Related Macular Degeneration [ANCHOR]), a retrospective analysis reported no significant differences in the rates of new choroidal neovascularization (CNV) in the nonneovascular fellow eyes of patients randomized to monthly ranibizumab at 12 and 24 months, compared with those randomized to sham injections (MARINA) or photodynamic therapy (ANCHOR) [12]. Similarly, a small prospective series of 26 patients with bilateral neovascular AMD treated with unilateral ranibizumab actually reported a significant increase in central retinal thickness (measured by OCT) in the fellow eye two weeks after injection [13].

However, a bilateral effect following unilateral anti-VEGF injection has been reported previously in some patients with neovascular AMD [3–5], DME [6–10], and uveitic cystoid macular edema [14]. For example, in a retrospective observational series of 25 patients with active neovascular AMD in one eye and disciform scarring in the fellow eye, treatment of the eye with active disease with ranibizumab resulted in clinical improvement in the fellow eye with disciform scarring, as measured by visual acuity, fluorescein angiography, and/or OCT [5]. Interestingly, one prospective study of 29 consecutive neovascular AMD patients treated with ranibizumab monthly for three doses reported a significant reduction in the central retinal thickness of the uninjected (nonneovascular) fellow eye [3].

The reason for the therapeutic effect in the untreated fellow eye is unknown but perhaps may be due to the presence of anti-VEGF agents in the systemic circulation [15]. In a prospective study, Avery et al. reported systemic exposure to ranibizumab, aflibercept, and bevacizumab along with associated reduced free VEGF levels, following intravitreal administration in 56 consecutive patients with neovascular AMD [16]. Significantly reduced levels of systemic VEGF were also reported after intravitreal injection of aflibercept and bevacizumab in 14 infants with retinopathy of prematurity [17]. The disruption of the blood-retina barrier in diabetes may enhance this effect [18].

## 4. Conclusion

Here, three such cases are reported in patients with neovascular AMD. These are nonconsecutive, nonrandomized patients who were noted to have this effect. The incidence of this effect is unknown but most likely rare. As further cases are reported, our understanding of this phenomenon should increase.
